# Targeting of viral interleukin-10 with an antibody fragment specific to damaged arthritic cartilage improves its therapeutic potency

**DOI:** 10.1186/ar4613

**Published:** 2014-07-16

**Authors:** Chris Hughes, Angelica Sette, Michael Seed, Fulvio D’Acquisto, Antonio Manzo, Tonia L Vincent, Ngee Han Lim, Ahuva Nissim

**Affiliations:** 1 Centre for Biochemical Pharmacology, William Harvey Research Institute, Barts and The London School of Medicine and Dentistry, Queen Mary University of London, London EC1M 6BQ, UK; 2 Medicines Research Group, School of Health Sport and Bioscience, University of East London, Water Lane, London E15 4LZ, UK; 3 Rheumatology and Translational Immunology Research Laboratories (LaRIT), Division of Rheumatology, IRCCS Policlinico San Matteo Foundation/University of Pavia, Pavia, Italy; 4 Kennedy Institute of Rheumatology, NDORMS, University of Oxford, 65 Roosevelt Drive, Headington, Oxford OX3 7FY, UK

## Abstract

**Introduction:**

We previously demonstrated that a single-chain fragment variable (scFv) specific to collagen type II (CII) posttranslationally modified by reactive oxygen species (ROS) can be used to target anti-inflammatory therapeutics specifically to inflamed arthritic joints. The objective of the present study was to demonstrate the superior efficacy of anti-inflammatory cytokines when targeted to inflamed arthritic joints by the anti-ROS modified CII (anti-ROS-CII) scFv in a mouse model of arthritis.

**Methods:**

Viral interleukin-10 (vIL-10) was fused to anti-ROS-CII scFv (1-11E) with a matrix-metalloproteinase (MMP) cleavable linker to create 1-11E/vIL-10 fusion. Binding of 1-11E/vIL-10 to ROS-CII was determined by enzyme-linked immunosorbent assay (ELISA), Western blotting, and immune-staining of arthritic cartilage, whereas vIL-10 bioactivity was evaluated *in vitro* by using an MC-9 cell-proliferation assay. Specific *in vivo* localization and therapeutic efficacy of 1-11E/vIL-10 was tested in the mouse model of antigen-induced arthritis.

**Results:**

1-11E/vIL-10 bound specifically to ROS-CII and to damaged arthritic cartilage. Interestingly, the *in vitro* vIL-10 activity in the fusion protein was observed only after cleavage with MMP-1. When systemically administered to arthritic mice, 1-11E/vIL-10 localized specifically to the arthritic knee, with peak accumulation observed after 3 days. Moreover, 1-11E/vIL-10 reduced inflammation significantly quicker than vIL-10 fused to the control anti-hen egg lysozyme scFv (C7/vIL10).

**Conclusions:**

Targeted delivery of anti-inflammatory cytokines potentiates their anti-arthritic action in a mouse model of arthritis. Our results further support the hypothesis that targeting biotherapeutics to arthritic joints may be extended to include anti-inflammatory cytokines that lack efficacy when administered systemically.

## Introduction

The hallmark of rheumatoid arthritis (RA) is chronic synovial inflammation that results in progressive joint damage. The pathogenesis of the disease is characterized by autoimmunity, chronic inflammatory synovitis, and destruction of the cartilage and the adjacent joint tissues [1]. These pathogenic processes are due to an imbalance in the cytokine network, where pro-inflammatory cytokines, such as tumor necrosis factor (TNF)-α, IL-1β, and IL-6 are overexpressed in the RA joint [2]. Homeostatic regulatory mechanisms in turn result in increased production of anti-inflammatory cytokines, such as IL-10 and transforming growth factor (TGF)-β, but this is not sufficient to counter the pro-inflammatory cytokines produced [3].

With this in mind, two alternative therapeutic approaches have been investigated. One is to neutralize the pro-inflammatory cytokines, and the other is to increase the concentration of the anti-inflammatory cytokines. Systemic treatment with TNF-α-blocking reagents is now a standard treatment of patients with RA failing to respond to conventional disease-modifying anti-rheumatic drugs (DMARDs) [4]. However, increasing evidence suggests that inhibition of TNF-α is associated with increased risk of infections due to general immune-suppression [5,6]. Moreover, despite the established clinical efficacy of anti-TNF-α, a subset of patients (30% to 40%) does not respond or has a suboptimal clinical response to anti-TNF-α treatment [7].

Our hypothesis is that targeting of biologic drugs to the inflamed joint will result in high local concentrations and low systemic concentrations, increasing efficacy while minimizing side effects. Additionally, a lower total dose may be effective, thereby reducing the cost of treatment. Targeting could be achieved by the identification of an inflamed joint tissue specific marker. We hypothesized that the influx of infiltrating leukocytes consumes increased amounts of oxygen and thereby generates high levels of ROS [8]. In turn, the influx of ROS results in chemical posttranslational modification of major specific cartilage components such as CII, resulting in formation of ROS-CII. ROS-CII would therefore be present in inflamed joints, but not in healthy joints, and thus represents an inflamed joint-specific target. Targeting may therefore be achieved by an ROS-CII-specific antibody.

By using a phage display human antibody library, we have developed a panel of human single-chain fragment variable (scFv) that binds specifically to ROS-CII [9]. One of these clones, 1-11E, localizes specifically in arthritic joints of mice. Hence, 1-11E fused to the murine tumor necrosis factor receptor 2-Fc-fusion protein (mTNFR2-Fc), which would scavenge pro-inflammatory TNF-α, had an enhanced therapeutic effect on inflamed knee swelling compared with mTNFR2-Fc fused to the nonrelevant anti-hen egg lysozyme (HEL) scFv, (C7/mTNFR2-Fc), or mTNFR2-Fc alone.

The current study is built on the previous study with 1-11E/mTNFR2-Fc to extend the range of targeted therapeutics to include an anti-inflammatory cytokine, IL-10. IL-10 is a major anti-inflammatory cytokine that inhibits the production of Th1 cytokines, such as interferon-γ, Th17 cytokines, and IL-17 [10], while increasing production of IL-1R, soluble TNF receptors, and enhanced release of Th2 cytokines. IL-10 also blocks NF-κB activity in macrophages, decreasing the expression of major histocompatibility complex class II and co-stimulatory molecules, and the production of TNF-α, IL-6, and IL-1 [11]. Systemic treatment of mice with collagen-induced arthritis (CIA) with recombinant IL-10 was efficacious, whereas anti-IL-10 antibodies exacerbated disease [12]. Viral IL-10 (vIL-10) has attracted attention for therapy, as it lacks some immunostimulatory effects of IL-10, while retaining all of the immunosuppressive actions of human IL-10 (hIL-10) [13]. Indeed, native and vIL-10 on rabbit antigen–induced arthritis (AIA) demonstrated equal efficacy [14].

Interestingly, gene therapy for CIA with adenoviral vectors encoding vIL-10 had a negligible effect when administered systemically, but significantly reduced disease when delivered intra-articularly [15]. Unfortunately, trials of IL-10 in RA were disappointing [16]. Efficacy might be increased if IL-10 were delivered locally. However, due to the number and inaccessibility of many affected joints in RA, direct injection of proteins or gene therapy vectors is not a feasible option.

A step toward targeting IL-10 to arthritic joints was reported by Trachsel and Schwarger *et al.*[17,18]. They fused human monoclonal antibodies specific to markers of angiogenesis (L19) to IL-10, IL-2, or TNF-α. Although L19/IL-2 and L19/TNF-α treatment led to increased arthritic scores and paw swelling, the L19/IL-10 fusion protein displayed therapeutic efficacy, which was superior to the activity of IL-10 fused to an antibody of irrelevant specificity in the CIA mouse. This work, however, targets IL-10 to an angiogenic marker, which is not exclusive to the damaged joint tissue.

In this study, we describe the development of 1-11E/vIL-10 fusion protein and the improved therapeutic efficacy of IL-10 in mice with antigen-induced arthritis when targeted to arthritic joints by anti-ROS-CII scFv, 1-11E.

## Methods

### Expression of 1-11E/vIL-10 fusion

**Figure 1:**
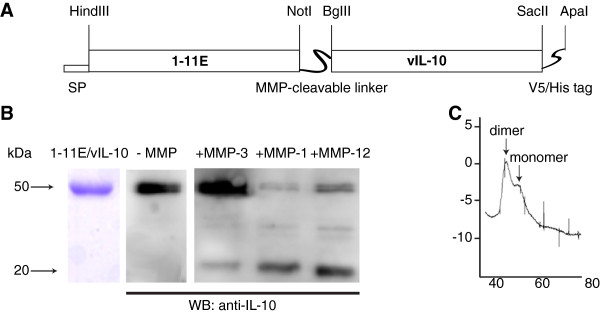
**Design and characterization of 1-11E/vIL-10 fusion. (A)** Schematic representation of the 1-11E/vIL10 fusion protein, as cloned into the pcDNA expression vectors. SP, signal peptide. The scFv and vIL-10 are separated by an MMP cleavage linker, whereas the His-tag and V5-tag were incorporated at the C-terminus. **(B)** SDS-PAGE analysis of purified 1-11E/vIL-10 is shown in the left panel. Purified 1-11E/vIL-10 fusion protein migrated at approximately 50 kDa, in keeping with the expected molecular weight (~30 kDa scFv plus ~20 kDa vIL-10). Digestion of 1-11E/IL10 by MMPs is shown in the right panel. 1-11E/vIL-10 fusion protein was incubated with the catalytic domains of MMP-1, MMP-3, and MMP-12 overnight and analyzed with Western blot by using anti-tetra-His-HRP antibodies. **(C)** FPLC profile of 1-11E/IL10. The first major peak at approximately 100 kDa corresponds to the dimer form, and the second minor peak at approximately 50 kDa corresponds to the monomer form.

The 1-11E/vIL-10 fusion was cloned into pcDNA6 vector (Invitrogen, Paisley, UK). vIL-10 was PCR with the following primers: Forward: 5′-AAAGCGGCCG CAGGGGGAGGCGGATCCCCGCTCGGGCTTTGGGCGGGAGGGGGCTCACAATGT GACAATTTTCCC-3′ and reverse: 5′TTTTGCGGCCGCCCTGGCTTTAATTGTCAT-3′. This resulted in vIL-10 omitted from its signal peptide at the 5′ end and replaced with the MMP cleavage site (PLGLWA) flanked by Gly_4_Ser_1_ linker from both sides (Figure 1A). After cloning IL-10 into the *Not*I and *Sac*II restriction sites, the scFv was amplified to contain the TNFR2 signal peptide (MAPAALWVALVFELQLWATGHT): forward: 5′-ATATATAAGCTT ATGGCGCCCGCCGCCCTCTGGGTCGCGCTGGTCTTCGAACTGCAGCTGTGGGCCACCGGGCACACATCTAGAATGGCCGAGGTGCAGCTG-3′, and reverse: 5′-ATATATGC GGCCGCCCGTTTGATTTCCACCTT-3′ and cloned into the *Hind*III and *Not*I. Similarly, hen egg lysozyme-specific scFv, C7, was cloned as fusion to vIL-10 (C7/vIL10) as nonrelevant scFv for negative control.

We performed transient transfections by using adherent HEK-293 cells using FuGENE 6 and according to manufacturer’s instructions (Promega UK, Southampton, UK). Culture medium was harvested after 3 days, and fusion protein was purified by using a nickel chelate column (QIAGEN, Crawley, UK) and following the manufacturer’s instructions.

### Evaluation of the molecular integrity of the fusion protein

Fusion protein was separated on SDS-PAGE under denaturing conditions after purification with Ni-NTA Agarose (Qiagen, Crawley, UK) or after overnight digestion of 1-11E/vIL-10 fusion by the catalytic domains of MMP-1, MMP-3, and MMP-12 [19]. Western blotting with anti-penta-His HRP was performed as described [9]. Gel filtration chromatography was performed by using a HiPrep 16/60 sephacryl S-200 (GE Healthcare, Little Chalfont, UK) column connected to an AKTA FPLC (GE Healthcare) in sterile 20 m*M* Tris–HCl, 150 m*M* NaCl, pH 8.

### *In vitro* bioactivity

Binding of purified fusion proteins to native CII and ROS-CII was determined by ELISA and Western blotting as described [9], with the bound fusion protein detected by using mouse anti-vIL-10 (R&D Systems, Abingdon, UK) followed by anti-mouse HRP (Sigma, Dorset, UK). Bioactivity of vIL-10 in the fusion protein was tested with a cell-proliferation assay by using mouse MC-9 mast cells as described [18]. In brief, on day 1, 5 × 10^4^ cells were treated with fusion proteins (with or without MMP-1 digestion) at concentrations of 1,000 ng/ml, 100 ng/ml, or 10 ng/ml, as well as control recombinant vIL-10 (R&D Systems, Abingdon UK). On day 4, cell viability was measured with Cell Titer Glo (Promega, Southampton, UK), and plates were read in a luminometer (Dynex, Worthing, UK) as per manufacturer’s instructions.

### Cartilage immunohistochemistry

Immunohistochemistry, by using damaged arthritic cartilage from several mouse models of arthritis, was performed. Samples were from C57BL/6 mice with antigen-induced arthritis (AIA) [20], DBA mice with collagen-induced arthritis (CIA) [19], and C57BL/6 with destabilization of the medial meniscus (DMM) [21]. All animal procedures were carried out under Home Office project licenses issued under the Animals (Scientific Procedures) Act 1986 as amended, and the institutional Animal Welfare and Ethical Review Bodies of Queen Mary University of London and University of Oxford, approved by the licenses from the Home Office. Immunohistochemistry was also performed by using human OA cartilage obtained from a patient undergoing prosthetic knee replacement (provided by Professor C. Montecucco, Fondazione IRCCS Policlinico S. Matteo, Pavia, Italy). Human OA were collected after consent, in accordance with institutional ethics policies and regulations (approved by Fondazione IRCCS Policlinico San Matteo, Pavia, Italy).

Safranin O staining was performed according to standard protocols [22]. Cartilage immunostaining was performed on 5-μm-thick sections, which were deparaffinized, hydrated, antigen retrieved, and blocked, as described [9]. Sections were incubated overnight at 4°C with 1-11E/vIL-10 or control C7/vIL-10 fusion (10 μg/ml) in DAKO diluent solution. Binding of the fusion protein was probed by using mouse anti-vIL-10 (R&D Systems, Abingdon, UK) followed by 1:1,000 anti-mouse HRP (Sigma, Dorset, UK). DAB substrate was used as peroxidase substrate (DAKO, Ely, Cambridgeshire, UK). Sections were counterstained with hematoxylin and mounted with DPX mount (BDH, London, UK). Fluorescent immunohistochemistry was also performed by using Cy5.5-labelled 1-11E/vIL-10 and C7/vIL-10. Cy5.5 labeling was done according to the manufacturer’s instructions (GE Healthcare, Buckinghamshire, UK). The pericellular matrix was stained by using the rat anti-heparan sulfate proteoglycan antibody (Millipore, Watford, UK) followed by the Alexa-Fluor-488 labeled anti-rat IgG (Life Technologies, Paisley, UK). Slides were viewed under the LSM 510 Meta (Zeiss, Cambridge, UK) using the 488-nm excitation laser to visualize the Alexa-488 label (green) and the 633-nm excitation laser to visualize the Cy5.5 label (red).

### Mouse models of arthritis

Female C57BL/6 mice of 10 weeks of age were used for AIA, as described previously [20]. In brief, after initial immunization with 100 μg mBSA in complete Freud adjuvant, inflammation was induced in the knees by intraarticular injection of 50 μg mBSA in PBS.

CIA was induced as described [19]. In brief, 10-week-old male DBA/1 mice were immunized by intradermal injection of an emulsion of 200 μg of bovine type II collagen in 100 μl of Freund complete adjuvant into the base of the tail.

Surgical destabilization of the medial meniscus (DMM) model of osteoarthritis was performed on 10-week-old C57BL/6 male mice, as described [23]. In brief, the right meniscotibial ligament was transected, resulting in the release of the medial meniscus from its tibial attachment.

### *In vivo* localization of 1-11E/vIL-10 in AIA

1-11E/vIL-10 and the control C7/vIL-10 were labeled with Cy5.5 according to the manufacturer’s instructions (GE Healthcare, Buckinghamshire, UK), resulting in a dye-to-protein ratio of 2.2. Before injecting the tagged fusion protein into mice, its integrity was first assessed with ELISA and immunohistochemistry, as described earlier. To track the fusion proteins *in vivo* after inflammation, 1 μg of Cy5.5-labeled fusion protein was injected intraperitoneally (i.p.) 1 day after the mBSA re-challenge in the AIA model. Epifluorescence images were obtained daily after anesthesia induction with isofluorane in an IVIS Spectrum imager, by using an excitation wavelength of 675 nm and an emission wavelength of 720 nm (Perkin Elmer, Waltham, MA, USA). Images were analyzed by using Living Image 4.4 to obtain the average fluorescence intensities of a circular region of interest encompassing the knee joint. Four days after i./p. injection of Cy5.5-labeled fusion protein, a single animal was killed and the knees, liver, kidney, heart, and spleen imaged *ex vivo*. The knees were further embedded in optimal-cutting-temperature media (VWR, Leicestershire, UK), before freezing in isopentane cooled by liquid nitrogen, before storage overnight at -80°C. The joints were sectioned at a thickness of 15 μm by using a Leica CM1900UV cryotome (Leica Biosystems, Milton Keynes, UK). The cryosections were fixed in ice-cold acetone before being air dried and blocked in DAKO blocking solution (DAKO, Ely, Cambridgeshire, UK). Fluorescence confocal microscope images of the sections were obtained, as described earlier.

### Treatment of AIA with 1-11E/vIL-10

On days 1 and 3 after the mBSA rechallenge in the AIA model, animals were injected i.p. with 30 μg of 1-11E/IL10 or C7/vIL-10. Swelling of the knee was measured daily by using calipers. On day 3, three animals were killed by cervical dislocation, serum was collected, and the knee joints were dissected and fixed in formalin (2% (vol/vol)) overnight, decalcified in EDTA for 5 weeks, and embedded in paraffin. Serial sections 2 μm in thickness were cut and further stained with safranin O. Serum cytokine concentrations were determined by using a seven-plex mouse pro-inflammatory assay kit (Mesoscale Discovery, Gaithersburg, MD, USA) in triplicate, according to the manufacturer’s instructions.

Levels of inflammation and redox state were determined by monitoring the luminescence after i.p. administration of 100 μl of 50 mg/ml luminol (5-amino-2,3-dihydro-1,4-phthalazine-dione; Sigma, Dorset, UK) in PBS. Luminol is a redox-sensitive compound that emits blue luminescence (λ_max_ 425 nm) when exposed to ROS but depends on myeloperoxidase activity [17]. The luminescence was determined by analyzing images obtained 15 minutes after luminol injection in the IVIS Spectrum Bioluminescence settings.

Safranin O-stained knee sections were scored for disease as follows: 0, Control; 1, no subsynovial inflammation, synovial healthy chondrocytes; 2, some evidence of soft-tissue edema and subsynovial inflammation, some zones of partial-thickness loss of cartilage staining; 3, marrow involvement becoming apparent, frank soft-tissue edema, moderate inflammation with synovial thickening, inflammatory tissue encroaching into joint, nonadhered. Cartilage matrix depletion evident; no chondrocyte death; 4, Edema of soft tissue, subsynovial inflammation, pannus encroachment and adhesion to cartilage, full-thickness cartilage staining depletion, chondrocyte clumping and death, and inflamed marrow.

### Statistical analysis

Statistical analyses were undertaken by using Prism (Graphpad, La Jolla, CA, USA) by using Newman-Keuls multiple-comparisons test. An α value of 0.05 was used as the threshold for significance.

## Results

### Biochemical analysis of fusion proteins

A schematic representation of 1-11E/vIL-10 fusion proteins is shown in Figure 1A. In SDS-PAGE analysis, purified 1-11E/vIL-10 fusion proteins migrated at approximately 50 kDa, in keeping with the expected molecular weight (~30 kDa scFv plus ~20 kDa IL-10, Figure 1B, left panel). When fusion proteins were incubated with MMP-1 or MMP-12, the C-terminal vIL-10 (~20 kDa) product was observed as a result of MMP-1 and MMP-12 cleavage but not with MMP-3 (Figure 1B, right panel). The molecular integrity of the 1-11E/vIL-10 fusion in solution was further demonstrated by gel-filtration chromatography, showing a major peak at 100 kDa, corresponding to the fusion protein dimer, anticipated as native IL-10 forms dimers. A smaller peak around 50 kDa corresponding to the fusion-protein monomer was also observed (Figure 1C).

### *In vitro* bioassay of 1-11E/vIL-10 fusion

**Figure 2:**
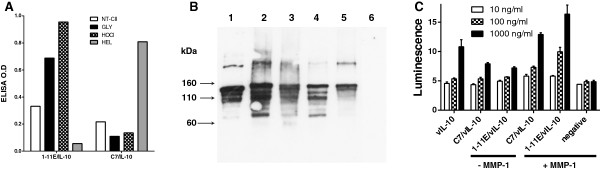
**Bioassay of 1-11E/vIL-10. (A)** ELISA showing increased binding of 1-11E/vIL-10 to ROS-CII (CII modified by glycation (Gly) or HOCl) compared with native CII (NT-CII). No binding to hen-egg lysozyme (HEL) was observed. In contrast, C7/vIL-10 bound only to HEL. **(B)** Western blot analysis showed that 1-11E/vIL10 bound to native CII (Lane 1), ROS modified CII (lane 2, glycated; lane 3, HOCl; lane 4, OH^-^; lane 5, peroxynitrate) but not to HEL (lane 6). **(C)**. IL-10 bioassay of 1-11E/vIL10 and C7/vIL10. We added 10, 100, or 1,000 ng/ml of fusion protein (white, pattern, and black boxes, respectively) to IL-10-responsive MC-9 cells with (+) or without (-) previous MMP-1 digestion. As a positive control, 10, 100, or 1,000 ng/ml commercial recombinant vIL-10 (vIL-10) was used, whereas MMP-1 alone-treated cells were used as a negative control. After 3 days, cell growth was measured with the Cell Titer Glo assay. Significant enhanced cell growth of the fusion proteins was observed after MMP-1 digestion (*P* < 0.01 for 1,000 ng/ml). No significant difference in growth was seen between 1-11E/vIL-10, C7/vIL-10, and vIL-10 (*P* > 0.05).

The antigen specificity of 1-11E/vIL-10 fusion proteins was determined with ELISA, by using native CII (NT CII), ROS-CII (CII modified by glycation (GLY) and HOCl (HOCl)), or control HEL as target antigens. 1-11E/vIL-10 had increased binding to both GLY and HOCl-derived ROS-CII (as previously demonstrated for 1-11E scFv [9]) and not to HEL (Figure 2A). Conversely, the C7/vIL-10 fusion protein was specific to HEL (Figure 2A).The specificity of the 1-11E/vIL-10 fusion to ROS-CII was further confirmed with Western blot. 1-11E/vIL-10 bound to all forms of CII, but not to HEL (Figure 2B). Binding pattern included binding to a range of CII fragments in the region of 25 to 100 kDa; and high-molecular-weight aggregates (higher than 250 kDa). In addition, 1-11E/vIL-10 bound to the electrophoretic band that corresponds to the intact native CII-chain polypeptide, although in ELISA, 1-11E/vIL-10 did not bind to native CII (Figure 2B).

The bioactivity of vIL-10 in the fusion protein was tested by its effect on MC-9 cell proliferation. Different concentrations (1,000, 100, and 10 ng/ml) of 1-11E/vIL-10 fusion proteins and C7/vIL-10 stimulated weak cell growth, but proliferation was increased (*P* < 0.01) up to comparable levels with commercial recombinant IL-10 after digestion with MMP-1(*P* > 0.05 for 1-11E/vIL-10 and C7/vIL-10 versus rIL-10). MMP-1 alone, however, was not able to induce any growth (*P* < 0.01, Figure 2C).

### Binding of damaged arthritic cartilage by 1-11E/vIL-10 fusion

**Figure 3:**
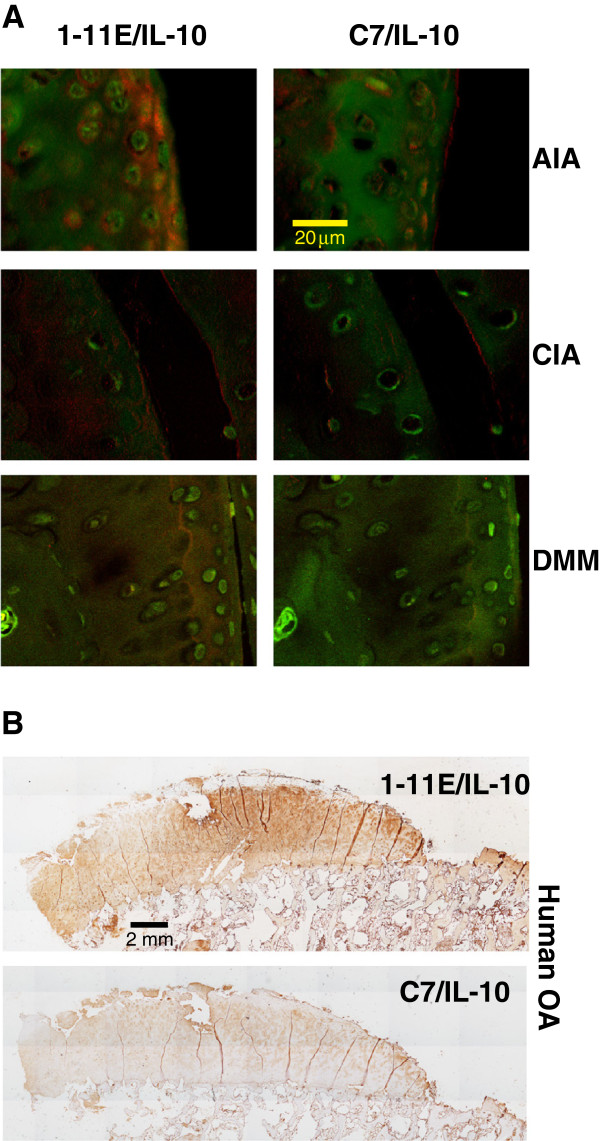
**Binding of 1-11E/vIL-10 to arthritic cartilage. (A)** Sections of cartilage from mouse model of antigen-induced arthritis (AIA), collagen-induced arthritis (CIA), and surgical destabilization of the medial meniscus (DMM) were incubated with Cy5.5 tagged 1-11E/vIL-10 (left panels) or C7/vIL10 (right panels). Binding to cartilage was diffused in all zones, although AIA staining was mostly in the superficial zone. No staining with C7/vIL10 was seen. Pericellular matrix of the chondrocytes is shown in green (AlexaFluor-488-labeled secondary) and the fusion protein (Cy5.5) in red. **(B)** Staining of human OA cartilage with 1-11E/vIL-10. Binding of the fusion protein was detected with mouse anti-vIL10, followed by anti-mouse-HRP. Binding was strong in the damaged area and as a territorial “halo” around the chondrocyte. Low background staining with C7/vIL-10 was seen.

We tested the binding specificity of 1-11E/vIL-10 to ROS-CII within the cartilage matrix of arthritic damaged cartilage from all three mouse models of arthritis (AIA, CIA, and DMM) and human OA cartilage by immunohistochemistry (Figure 3). In AIA cartilage, a diffuse and strong pattern of staining with 1-11E/vIL-10 of the artificial zone was observed. No staining was seen with C7/vIL-10. A similar pattern of staining was seen for CIA cartilage. The staining of DMM cartilage was more diffuse, and we observed staining in all zones (superficial, middle, and deep zone). No background staining was seen for control C7/vIL-10 (Figure 3A). No staining of healthy mouse cartilage was observed (data not shown). Human OA cartilage displayed a diffuse pattern of staining as a territorial “halo” around the chondrocyte with 1-11E/vIL-10 and with very strong staining in extensive eroded areas as a reflection of high levels of ROS-CII in these areas (Figure 3B). Very low background staining with C7/vIL-10 was seen in human cartilage around the chondrocytes.

### *In vivo* localization of 1-11E/vIL-10 fusion to inflamed tissue in AIA

**Figure 4:**
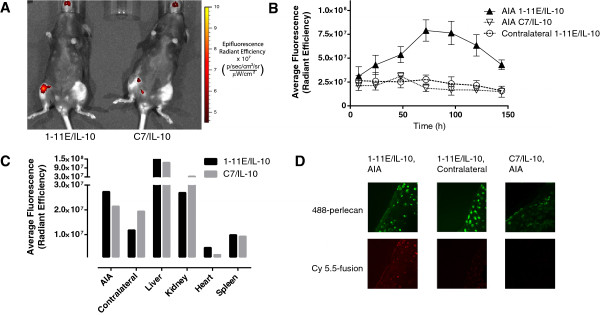
**Tracking of 1-11E/vIL-10 to arthritic joints.** One day after AIA induction in mice, 1 μg of Cy5.5-labeled fusion proteins was injected i.p. *In vivo* fluorescence images of the mice were taken to follow the tracking of the fusion proteins to the joint. **(A)** Representative fluorescence image taken 3 days after injection of 1-11E/vIL-10 (left) and C7/vIL-10 (right). **(B)** Quantification of the average fluorescence of the region of interest encompassing the knee joint, *n* = 3. **(C)***Ex vivo* quantification of fluoresce of tissues after dissection of a single mouse 4 days after injection. **(D)** Representative fluorescence images of cryosections of the excised knee joints, showing the pericellular matrix of the chondrocytes in green (AlexaFluor-488-labeled secondary) and the fusion protein (Cy5.5) in red.

The targeting function of the 1-11E portion of the fusion protein to inflamed tissue was addressed by tagging the fusion protein with the Cy5.5 fluorophore and injecting i.p. into animals with AIA. Subsequent *in vivo* fluorescence imaging indicated that the 1-11E/vIL-10 fusion protein selectively tracked to the inflamed joint, compared with the contralateral uninflamed joint and the control C7/vIL-10 fusion protein (representative images taken 3 days after i.p. injection shown in Figure 4A).

Quantification of the average fluorescence within a circular region of interest encompassing the knee joint indicated that the maximum amount of 1-11E/vIL-10 fusion protein tracking to the inflamed joint occurred 3 days after i.p. injection (Figure 4B; *P* < 0.0001). *Ex vivo* fluorescence imaging of the internal organs 4 days after i.p. injection indicated no difference in the clearance of 1-11E/vIL-10 and C7/vIL-10 (Figure 4C), demonstrated by strong florescence seen in the kidney and liver because of clearance. Nevertheless, no signal was seen in the heart and spleen. Frozen sections of excised knee cartilage indicate that the Cy5.5-labeled 1-11E/vIL-10 is present throughout the cartilage of inflamed joints, as well as within some chondrocytes, but not in the contralateral joint (Figure 4D). C7/vIL10 did not show staining of the excised cartilage.

### Therapeutic efficacy of 1-11E/vIL-10 fusions in mouse model of arthritis

**Figure 5:**
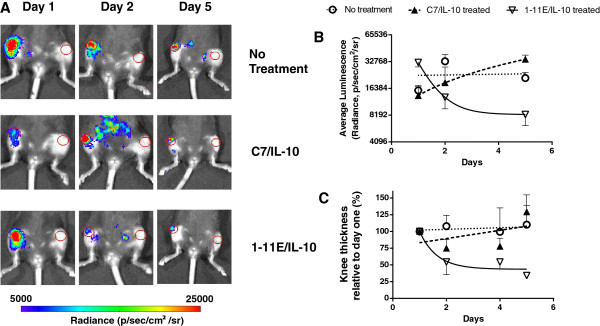
**Reduction of oxidative state in inflamed knee by 1-11E/vIL10 fusion proteins.** Antigen-induced arthritis was induced in female C57BL/6 mice by injecting mBSA into the knee of each animal 1 day before treatment (*n* = 3). On days 1 and 3 after mBSA rechallenge, animals were injected i.p. with 30 μg of 1-11E/vIL10 or C7/vIL10. As a control, PBS was injected. Level of inflammation and redox state were determined by monitoring the luminescence 15 minutes after i.p. administration of luminol, a redox-sensitive compound that glows when mixed with oxidizing agents present in the inflamed knee and thus correlates with degree of inflammation. **(A)** Representative luminescence images taken at days 1, 2, and 5 after injection of 1-11E/vIL-10, C7/vIL-10, and control nontreated mice are shown. **(B)** The reduction in oxidation level was faster in the 1-11E/vIL-10-treated animal than in C7/vIL-10 and nontreated mice, which correlated with reduction in swelling **(C)**.

The therapeutic potency of the fusion proteins was tested in two consecutive sets of experiments by using the murine AIA arthritis model in C57BL/6 mice. After rechallenge with mBSA, mice with a similar degree of knee swelling were selected for treatment. In the first experiment, a small group of animals (*n* = 3) was injected i.p. with 30 μg 1-11E/vIL-10 or control C7/vIL-10 after mBSA rechallenge to determine the ability of 1-11E/vIL-10 treatment to reduce oxidative stress as a result of the inflammation. We measured the level of oxidants present in the inflamed knee by i.p. injection of luminol. Figure 5A shows that the reduction in levels of luminescence was accelerated in 1-11E/vIL-10 treated mice compared with mice treated with C7/vIL-10 or nontreated control mice. Hence, the intensity of the luminol signal in the arthritic knee correlated with accelerated reduction of knee swelling by 1-11E/vIL-10 in comparison to C7/vIL-10 and control nontreated mice (Figure 5B, C, *p* = 0.6317).

**Figure 6:**
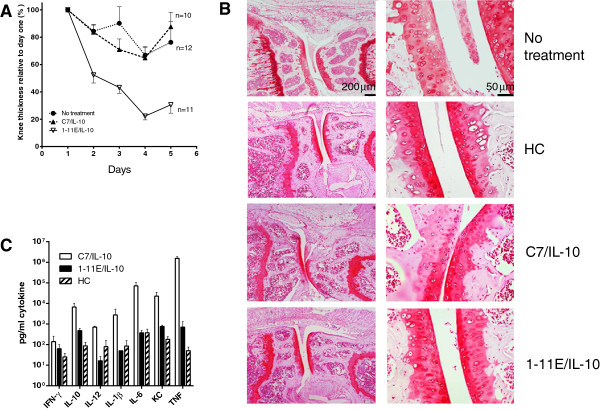
**Treatment efficacy of 1-11E/vIL-10. A**. AIA was induced in female C57BL/6-mice 1 day before treatment (*n* = 10-12). On days 1 and 3 after mBSA rechallenge, animals were injected i.p. with 30 μg of 1-11E/vIL10 or C7/vIL10 or PBS. Significant reduction in knee swelling in 1-11E/vIL-10 treated mice compared with mice treated with C7/vIL-10 or PBS controls was observed (*P* = 0.0048). **(B)** Safranin O staining of cartilage from nontreated AIA knee displayed weak staining. The cartilage from healthy control animals was smooth, with clear strong staining with safranin O. Cartilage from C7/vIL-10-treated mouse had weak safranin O staining compared with healthy controls. 1-11E/vIL-10-treated knees displayed histology similar to that of the healthy joints. **(C)** Serum cytokine levels in treated mice were obtained from mice treated with 1-11E/vIL10 or C7/vIL10 as well as from healthy control animals (HC) on day 3. Cytokines levels were measured with ELISA by using the 7-plex mouse Pro-Inflammatory Cytokine Kit. Except for IFN-γ, levels of pro-inflammatory cytokines were significantly lower in the 1-11E/vIL-10-treated animal (*P* = 0.0175) and similar to levels of healthy control animals (*P* > 0.05).

To confirm further the efficacy of treatment with 1-11E/vIL-10, we performed a large experiment using AIA mice (*n* = 10-12) injected with 30 μg per injection of the 1-11E/vIL-10 or control C7/vIL-10 fusion protein at days 1 and 3 after rechallenge with mBSA. Once again, we observed a significant reduction in knee swelling in 1-11E/vIL-10 treated mice compared with mice treated with C7/vIL-10 or PBS controls (Figure 6A).

Histologic analysis of the excised knee joints from 1-11E/vIL-10-treated versus C7/vIL-10-treated mice from day 3 showed a clear difference between the two groups (Figure 6B). In joints from the nontreated mice, several pathologic features of a moderate arthritis were observed. Edema of the soft tissues was noted, with thickening of the subintima with a vigorous inflammatory infiltrate. Encroachment of inflammatory pannus from the point of insertion was found, with a thin layer of inflammatory tissue adhering and overlying the cartilage associated with roughening. Cartilage was heavily depleted of glycosaminoglycan staining, with some islands of new synthesis surrounding chondrocytes. Evidence of chondrocyte clumping was found, and some lacunae were absent of chondrocytes. Bone marrow was inflamed, and subchondral bone depleted and disorganized (Score 4). The cartilage from healthy control (HC) animals was smooth, with clear staining with safranin O (Score 0). Treatment with C7/vIL-10 showed a reduced inflammatory picture compared with nontreated joints. Bone marrow inflammation was absent, subchondral bone healthy, and cartilage showed much stronger glucosaminoglycan staining. However, zones of cartilage glycosaminoglycan depletion were still evident. Soft-tissue edema was reduced, but not to the levels of healthy control, and some inflammatory infiltrate with synovial lining layer thickening was observed (Score 2). Treatment with 1-11E/vIL-10 looked essentially normal. Although some sublining layer inflammatory influx was noted, very little thickening occurred. The synovial lining layer looked normal, with no encroachment from the point of insertion. Cartilage appeared healthy, smooth, and normal, with staining for glycosaminoglycan equivalent to that of the healthy control, with no patches of depletion, as seen in the C-7/IL10-treated joint. Marrow and subchondral bone appeared normal (Score 0). Cytokine profiles were analyzed in three animals from each group on day 3 (Figure 6C). Pro-inflammatory cytokines levels in the 1-11E/vIL-10-treated group were significantly lower than in the C7/vIL-10 group (*P* = 0.0175, except for IFN-γ, which was similar in all groups) and similar to the levels observed in the healthy control animals.

## Discussion

Biological therapies have revolutionized treatment for RA patients; however, they have various drawbacks due to incomplete response in 30% to 40% of patients, side effects, and high cost [24]. Development of novel approaches to overcome these limitations is becoming even more important, as recent evidence supports the advantage of earlier use of TNF-α inhibitors, before the failure of conventional DMARDs, and especially to use TNF inhibitors in combination with methotrexate as first-line therapy in patients with poor prognosis and signs for rapidly progressive disease [25]. Our hypothesis is that targeted therapy may address at least some of these drawbacks, as it allows concentration of the bioactive molecules within the damaged joints and, thus, increase potency while minimizing side effects. In addition, targeted therapy may resolve some of the obstacles in achieving beneficial treatment with anti-inflammatory cytokines, as opposed to treatment by blockade of pro-inflammatory cytokines, which has become an established treatment for RA. Because of the poor pharmacokinetics (~30 minutes half-life) of anti-inflammatory cytokines, high nontolerable dosages have been used to achieve efficacy in clinical trials. In fact, the clinical development of both interferon β [26,27] and IL-10 [16] was discontinued because of an insufficient efficacy.

Here we describe that vIL-10 fused to the human antibody fragment specific for damaged arthritic cartilage is a valid targeted anti-inflammatory therapy in the treatment of a mouse model of arthritis.

Although the original anti-ROS-CII specificity of 1-11E was maintained in 1-11E/vIL-10, the *in vitro* vIL-10 bioactivity of 1-11E/vIL-10 and C7/vIL-10 fusion proteins was very low until they were cleaved by MMP-1, indicating that vIL-10 was latent before the MMP-1 digestion. Human IL-10 (hIL-10) fused to the scFv was, however, active [17,18], raising the very interesting question of the difference between hIL-10-and vIL-10 as a fusion partner in terms of therapeutic potential. vIL-10 has been shown to bind to and signal through human IL-10R1 [28]. The regions on the surfaces of the hIL-10 and vIL-10 that make contact with the receptor are essentially the same. The binding affinity of vIL-10 (which has 92% sequence identity to hIL-10) to cell-surface IL-10R1 is, however, ~1,000-fold lower than that of hIL-10 [29]. This difference in receptor-binding affinity is thought to be caused by subtle changes in the conformation and dynamics of two loop structures and the interdomain angle [30] as a result of a single amino acid substitutions at position 87 of isoleucine to alanine (I87A) in vIL-10 [29]. Similar to vIL-10, I87A substitution in hIL-10 results in hIL-10 with only immunosuppressive, but not immunostimulatory functions. Keravala *et al*. [14] demonstrated that the I87A mutant resulted in significant improvement of the pathology in the treated joints to similar levels as the vIL-10, whereas Ding *et al.*[31] showed that the I87A mutant induced similar Stat1, Stat3, and Stat5 activation as hIL-10. The latency of vIL-10 in our fusion probably reflects the low affinity of vIL-10 in comparison to hIL-10, where we used 1,000 ng of 1-11E/vIL-10 and control vIL-10 to see significant growth effect on the MC-9 cells, whereas 10 ng of hIL-10 was sufficient [17]. The close proximity of the scFv probably induced further conformational changes as well as changes in the dynamics of two loop structures and the interdomain angle, which has resulted in lack of binding to the receptor. The fact that 1-11E/vIL-10 is systemically latent until it is cleaved by MMP in the arthritic joints is advantageous, as it will be activated only in inflamed joints where local MMPs, especially MMP-12 in early inflammation [19], cleaves it from the fusion, with little sequestering of the cytokine by systemic receptors [32].

1-11E/vIL-10 maintained the specific binding to arthritic cartilage. Hence, the *in vivo* accumulation of 1-11E/vIL-10 in the inflamed joint showed a dramatic improvement compared with 1-11E scFv, with sustained targeting for 4 days compared with 2 hours, respectively. Possibly this is due to the increased molecular weight to 100 kDa for 1-11E/vIL-10 compared with 30 kDa for 1-11E scFv, and above the kidney threshold. This also explains why treatment efficacy was maintained for several days. The 1-11E/vIL-10 fusion greatly accelerated the reduction of knee-joint swelling, redox state, and levels of pro-inflammatory cytokines in comparison to control C7/vIL-10. The lack of therapeutic effect with the C7/vIL-10 could be explained by the lack of targeting to the arthritic joint, which then resulted in much lower (suboptimal) local concentration in the arthritic joints. Interestingly, serum levels of mouse pro-inflammatory cytokines in the 1-11E/vIL-10-treated mice were significantly lower compared with C7/vIL-10-treated mice and almost as in healthy mice. Mouse IL-10 levels in 1-11E/vIL-10-treated mice was, however, a bit lower than that observed in C7/vIL-10-treated mice, possibly reflecting the reduced inflammation in the 1-11E/vIL-10-treated group and thus fewer anti-inflammatory cytokines were needed to combat the inflammation. These data, however, must be further confirmed in a larger experimental setting.

## Conclusions

The results from this study demonstrate that anti-inflammatory cytokines can be targeted specifically to diseased arthritic joints with increased efficacy in comparison to nontargeted treatment. These data demonstrate a further milestone with potential for further development of optimal treatment of RA, where blockade of pro-inflammatory cytokines or treatment with anti-inflammatory cytokines may be used alone, or in combination. Indeed, the testing of combination biological therapies for RA has largely been avoided for safety concerns, but targeted therapeutics may enable this in the future.

## Abbreviations

1-11E: scFv specific to ROS-CII; AIA: antigen-induced arthritis; C7: scFv specific to HEL; CIA: collagen-induced arthritis; CII: collagen type II; DMM: destabilization of the medial meniscus model of osteoarthritis; ELISA: enzyme-linked immunosorbent assay; HEL: hen egg lysozyme; IHC: immunohistochemistry; MPP: matrix-metalloproteinase; ROS: reactive oxidants; ROS-CII: CII posttranslationally modified by ROS; scFv: single-chain fragment variable; vIL-10: viral interleukin-10.

## Competing interests

The authors declare that they have no competing interests.

## Authors’ contributions

CH, FD, TV, NHL, and AN made substantial contributions to conception, design, interpretation of data, and critical revision of the manuscript for intellectual content. CH, NHL, and AN drafted the manuscript. CH was involved in the cloning and characterization of the fusion proteins and the AIA model. AS carried out the expression of the fusion proteins, IHC, *in vivo* imaging, and interpretation of the imaging data. NHL was involved in the *in vivo* models, IHC, *in vivo* imaging, and imaging data analysis. MS and AM were involved in the acquisition, interpretation of the IHC experiments, and scoring of disease severity. All authors have given final approval of the version to be published.
